# Nurse-Led Care: A Case Report of Twin Pregnancy Complicated by Ureteral Calculus and Thyrotoxicosis

**DOI:** 10.3390/jcm15176910

**Published:** 2026-09-07

**Authors:** Qiong Yu, Rui Shi, Ying Li, Rong Na, Xinliang Cai

**Affiliations:** 1 Department of Urology, The University of Hong Kong-Shenzhen Hospital, Shenzhen 518000, China; 2 Department of Obstetrics and Gynecology, The University of Hong Kong-Shenzhen Hospital, Shenzhen 518000, China; 3 Department of Pharmacy, The University of Hong Kong-Shenzhen Hospital, Shenzhen 518000, China

**Keywords:** nurse-led care, urolithiasis, thyrotoxicosis, twin pregnancy, multidisciplinary care

## Abstract

**Background**: Managing urinary calculi during a multiple gestation pregnancy presents significant clinical challenges, which require rigorous care to prevent adverse maternal and fetal outcomes. This report aims to offer clinical nursing references by summarizing the management of a complex pregnancy complicated by ureteral calculi and thyrotoxicosis. **Case Presentation**: A patient with a twin gestation complicated by a ureteral calculus and thyrotoxicosis was managed with nurse-led continuous care within a physician-directed multidisciplinary framework. During hospitalization, interdisciplinary medical and nursing teams continuously monitored maternal and fetal vital signs to establish individualized surgical and nursing regimens. Post-discharge integrated care was delivered via online and offline follow-up, incorporating collaborative assessments from the obstetrics, endocrinology, and psychology departments, alongside multidimensional physical care and targeted psychological counseling. **Conclusions**: With standardized treatment and continuous nursing intervention, the patient delivered healthy boy-girl twins by caesarean section at 37 weeks of gestation, followed by successful ureteroscopic lithotripsy and uncomplicated hospital discharge. This case illustrates a feasible approach to nurse-led continuous care within a physician-directed multidisciplinary framework for complex, high-risk pregnancies. As this is a single case, the findings should be interpreted cautiously and require further validation.

## 1. Introduction

Urinary calculi are common and prevalent urological diseases worldwide. Epidemiological surveys have demonstrated that the prevalence of urinary calculi in China reaches 6.5% [1]. As a vulnerable population requiring rigorous clinical care, pregnant women possess unique physiological characteristics, and the incidence of gestational urinary calculi cannot be ignored. Previous studies have reported that the incidence of urinary calculi during pregnancy ranges from 0.2% to 0.49% [2,3]. Notably, small-sized calculi can be spontaneously excreted without obvious clinical manifestations, suggesting that the actual incidence may be substantially higher. While most gestational nephrolithiasis is asymptomatic and resolves spontaneously, uterine enlargement in the middle and late trimesters can compress the ureter, combined with gestational metabolic disorders, potentially leading to ureteral calculus incarceration. This condition may further induce severe complications, including urinary tract infection, hydronephrosis, renal colic, and even urosepsis and septic shock, which pose substantial threats to maternal and fetal safety. A population-based study involving 4.7 million participants confirmed that gestational nephrolithiasis is associated with an increased risk of preeclampsia, preterm birth, low birth weight, and a higher cesarean delivery rate [4]. Pregnancy is accompanied by several physiological changes that may influence urinary stone formation. Increased glomerular filtration and urinary excretion of calcium, uric acid, and other lithogenic substances may increase urinary supersaturation, whereas progesterone-mediated relaxation of ureteral smooth muscle and mechanical compression of the ureters by the enlarging uterus can promote urinary stasis and physiological hydronephrosis. Although increased urinary citrate may exert a protective effect, alterations in urinary pH and mineral excretion may disturb the balance between lithogenic and inhibitory factors. These interacting metabolic and anatomical changes may contribute to the development or clinical manifestation of urinary calculi during pregnancy [5]. Thyrotoxicosis represents another clinically relevant condition during pregnancy and the postpartum period. Uncontrolled thyrotoxicosis may cause persistent tachycardia, cardiovascular instability, and, in severe cases, thyroid storm, thereby increasing perioperative and maternal risks. When urinary tract disease and thyroid dysfunction coexist, treatment planning becomes more complex and requires coordinated assessment across urology, obstetrics, endocrinology, pharmacy, and nursing disciplines [6]. Therefore, continuous monitoring and multidisciplinary management are particularly important in such complex cases. Accordingly, ensuring the safety and quality of life of pregnant women with urinary calculi remains a critical priority in clinical obstetric care. Nurses play an indispensable role in the multidisciplinary management of high-risk pregnancies. Scientific assessment of maternal physical and psychological status and standardized, continuous nursing intervention are essential to optimizing clinical outcomes. Within a physician-directed multidisciplinary framework, nurse-led continuous care enables nurses to take charge of continuous assessment, patient education, care coordination, and follow-up for patients with complex comorbidities. Previous studies suggest that nurse-led services may contribute to continuity of care through structured assessment, patient education, care coordination, and follow-up [7]. In this study, we report a case of a second-trimester twin pregnancy complicated by ureteral calculi and thyrotoxicosis. The aim of this report is to describe the clinical course and multidisciplinary management of this complex pregnancy, with particular emphasis on the role of nurse-coordinated continuous care within a physician-directed multidisciplinary framework. The patient achieved a favorable clinical experience throughout treatment and observation and was discharged successfully. The clinical details and nursing experience are reported as follows.

## 2. Case Presentation

### 2.1. Case History

A 28-year-old female patient at 14 + 3 weeks of dichorionic diamniotic twin gestation presented to the emergency department with severe right lower abdominal colic lasting 12 h. She had an unremarkable medical history and no prior similar painful episodes. No fever or other discomforts were reported before admission. Vital signs upon presentation were temperature 37.1 °C, pulse 99 beats/min, respiratory rate 20 breaths/min, room-air oxygen saturation 99%, and blood pressure 125/90 mmHg. Twin fetal heart rates were consistent with normal gestational parameters.

### 2.2. Nurse-Led Care Model

Medical decision-making in this case, including the choice of conservative versus surgical management, drug selection, indications for ureteral stent placement, the antithyroid regimen, and the timing of definitive lithotripsy, was directed by physicians and the multidisciplinary team (MDT). Within this framework, nurses independently conducted symptom assessment, medication adherence education, lifestyle guidance, adverse-event screening, and routine follow-up. Nurses were authorized to reinforce education, adjust follow-up intensity, and provide individualized self-management advice according to the patient’s condition. Escalation to the physician or the MDT was initiated when persistent or worsening symptoms, abnormal laboratory findings, suspected adverse drug reactions, poor treatment adherence, or other clinically significant changes were identified. Stent-related symptoms were monitored with the Ureteral Stent Symptom Questionnaire (USSQ) [8], and anxiety was monitored with the anxiety subscale of the Hospital Anxiety and Depression Scale (HADS-A). All assessments reported here are serial self-reported measurements from this single patient.

### 2.3. Diagnostic Investigations

**Figure 1:**
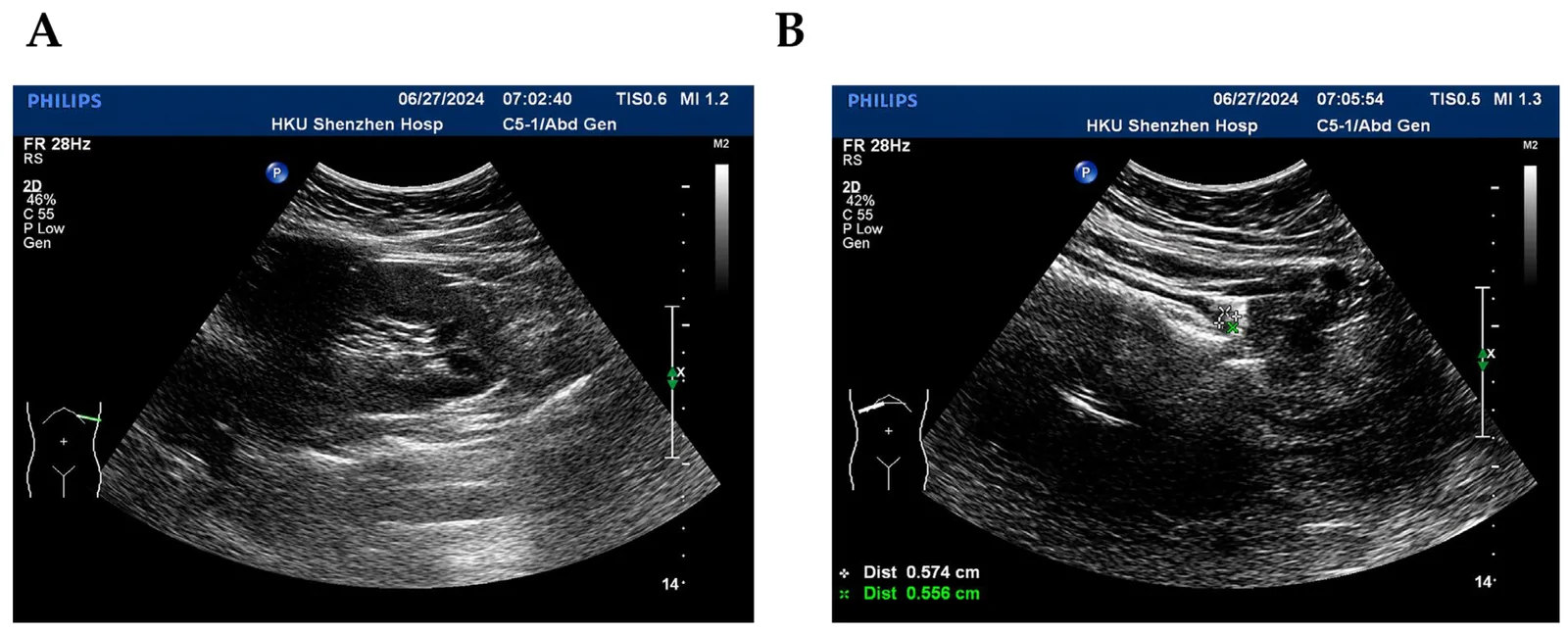
Urinary ultrasonography shows a right ureteral calculus with mild right hydronephrosis. The arrow indicates the 6 × 6 mm calculus located between the second and third anatomical constrictions of the right ureter. (**A**) Dorsal; (**B**) Abdomen.

**Figure 2:**
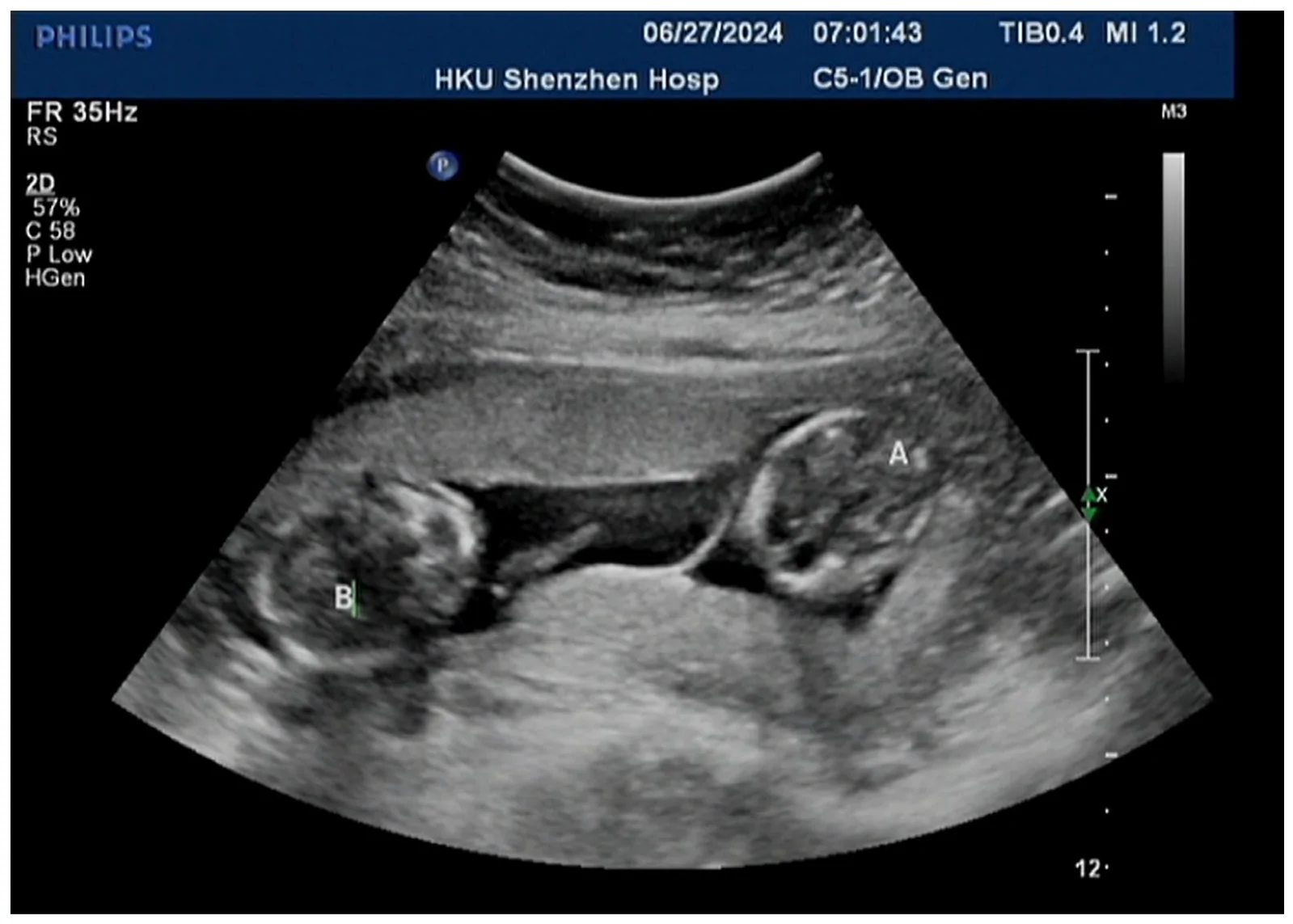
Ultrasonography shows two viable fetuses with normal amniotic fluid volume, indicating a twin pregnancy. “A” and “B” denote twin A and twin B.

Urinary ultrasonography identified a 6 × 6 mm calculus located between the second and third anatomical constrictions of the right ureter, complicated by mild right hydronephrosis (Figure 1). Prenatal ultrasound verified an intrauterine dichorionic diamniotic twin pregnancy with normal amniotic fluid volume (Figure 2). Laboratory examinations demonstrated an elevated white blood cell count of 15.82 × 10^9^/L (reference range 3.50–9.50 × 10^9^/L), with a C-reactive protein level of 3.0 mg/L (reference < 5 mg/L) and a serum creatinine level of 53 μmol/L (reference range 44–80 μmol/L), both within normal limits. Urinalysis demonstrated marked haematuria, with occult blood 3+ and a urinary sediment red blood cell count of 207.6/μL, together with 25 leucocytes/μL and proteinuria 1 + (0.3 g/L).

### 2.4. Treatment, Outcomes, and Follow-Up

Upon admission, the urological nursing team immediately initiated the standardized management protocol for pregnancy-complicated renal colic. Full-scale physical and psychological assessments, symptomatic analgesia, emotional soothing, and anti-infective therapy were performed. A detailed medical history was collected, and a multidisciplinary team (MDT) consultation was prepared accordingly. The patient’s pain was gradually relieved, and her tense and fearful mood was stabilized after initial interventions. Subsequently, the medical and nursing team thoroughly explained the disease condition and therapeutic regimens to the patient and her family. After full consideration, the patient initially opted for conservative medical treatment. However, recurrent renal colic attacks occurred twice within the subsequent 10 h, and the patient requested surgical intervention.

After repeated informed consent and detailed explanation of the surgical procedure, transurethral right ureteral stent placement was successfully performed under local anesthesia on the same day. The operation lasted 20 min with zero intraoperative bleeding. Ultrasound examination verified the accurate position of the indwelling ureteral stent. Preoperatively, nurses took the lead in continuous maternal vital sign monitoring and fetal heart surveillance, closely observing maternal reactions and fetal dynamic changes. Standardized medication management of antibiotics and analgesics was implemented to balance maternal comfort and medication safety.

Postoperatively, nurses actively coordinated MDT evaluation involving urologists, obstetricians, psychologists, ultrasonographers, and clinical pharmacists to jointly optimize the individualized treatment regimen. The patient exhibited a good mental state without recurrent pain. Urination was unobstructed after urinary catheter removal on the first postoperative day, with no signs of vesicoureteral reflux such as lumbago or distension during urination. After stent placement, the white blood cell count was 15.06 × 10^9^/L, with C-reactive protein 4.8 mg/L and serum creatinine 39 μmol/L; before discharge, the white blood cell count had decreased to 10.63 × 10^9^/L, with C-reactive protein 10.8 mg/L and serum creatinine 36 μmol/L. Renal colic and urinary symptoms resolved, and the patient was discharged on the second postoperative day.

**Figure 3:**
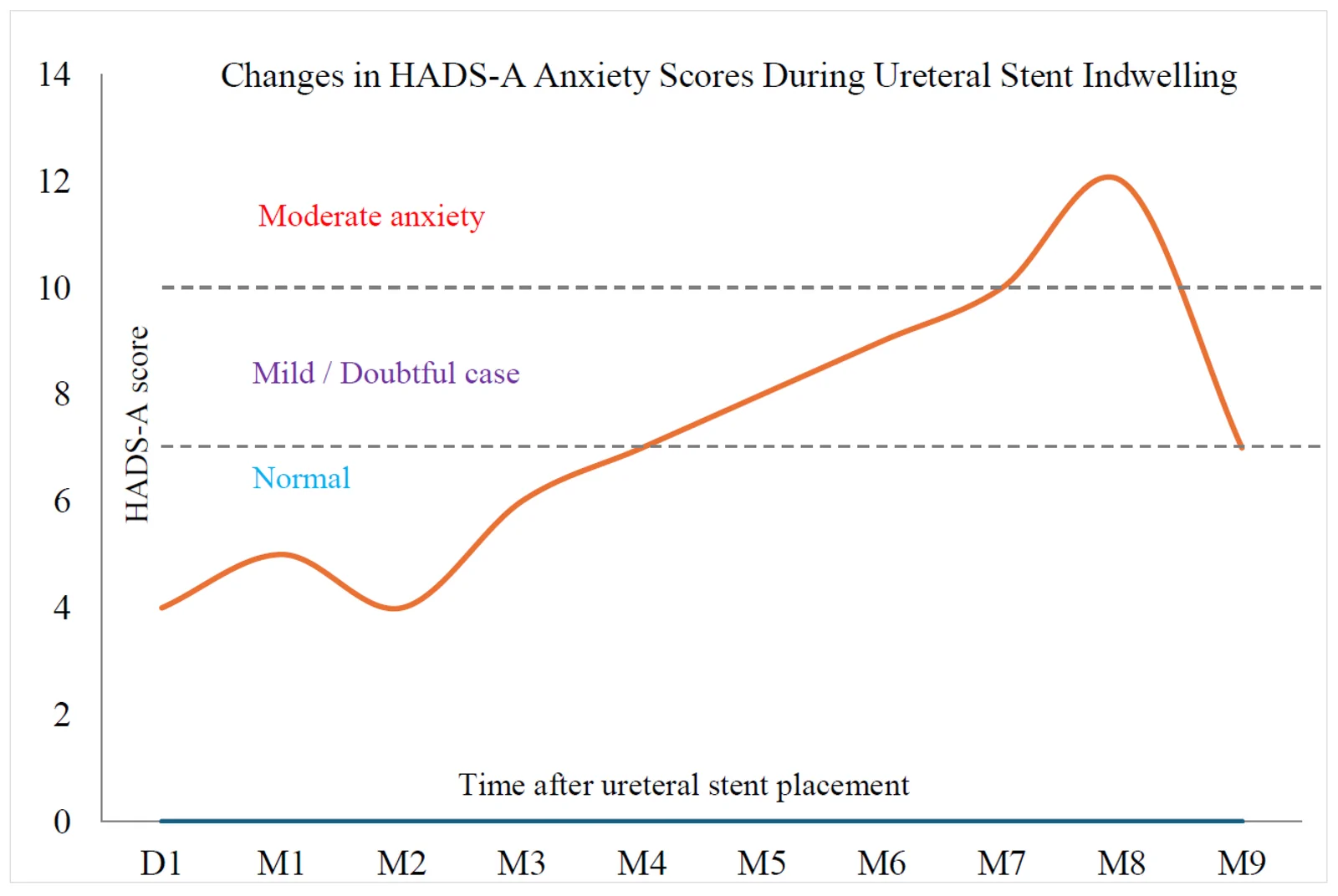
Nurse-led dynamic assessment of anxiety and multidisciplinary psychological intervention during ureteral stent indwelling. X axis: D1, day 1; M1–M9, months 1–9 after ureteral stent placement. Y axis: score on the anxiety subscale of the Hospital Anxiety and Depression Scale (HADS-A). Data are serial self-reported measurements obtained from a single patient.

After discharge, the nursing team implemented an internet-based continuing nursing model via online and offline follow-up. Regular health education was delivered through social platforms, including daily precautions for indwelling ureteral stents, guidance on adequate water intake and urine volume maintenance, and avoidance of urinary retention and reflux-induced infection. The patient was instructed to monitor body temperature and fetal heart rate regularly, complete monthly stent-related symptom and anxiety assessments, and attend regular follow-up visits in the departments of obstetrics and gynecology and urology. Nurses maintained continuous communication with multidisciplinary physicians to dynamically monitor and update the patient’s condition management plan. During follow-up, multidisciplinary communication was arranged according to the patient’s clinical needs. Nurses summarized symptom changes, treatment adherence, laboratory findings, and potential adverse events and communicated these findings to the responsible physicians when escalation criteria were met. Additional multidisciplinary discussion was initiated whenever clinically significant changes required treatment reassessment. Serial HADS-A assessments showed a gradual increase in anxiety symptoms during follow-up, with the score reaching its highest level at Month 8, coinciding with the diagnosis and treatment of thyrotoxicosis. Following endocrine treatment and targeted psychological counselling, the HADS-A score subsequently decreased (Figure 3).

**Figure 4:**
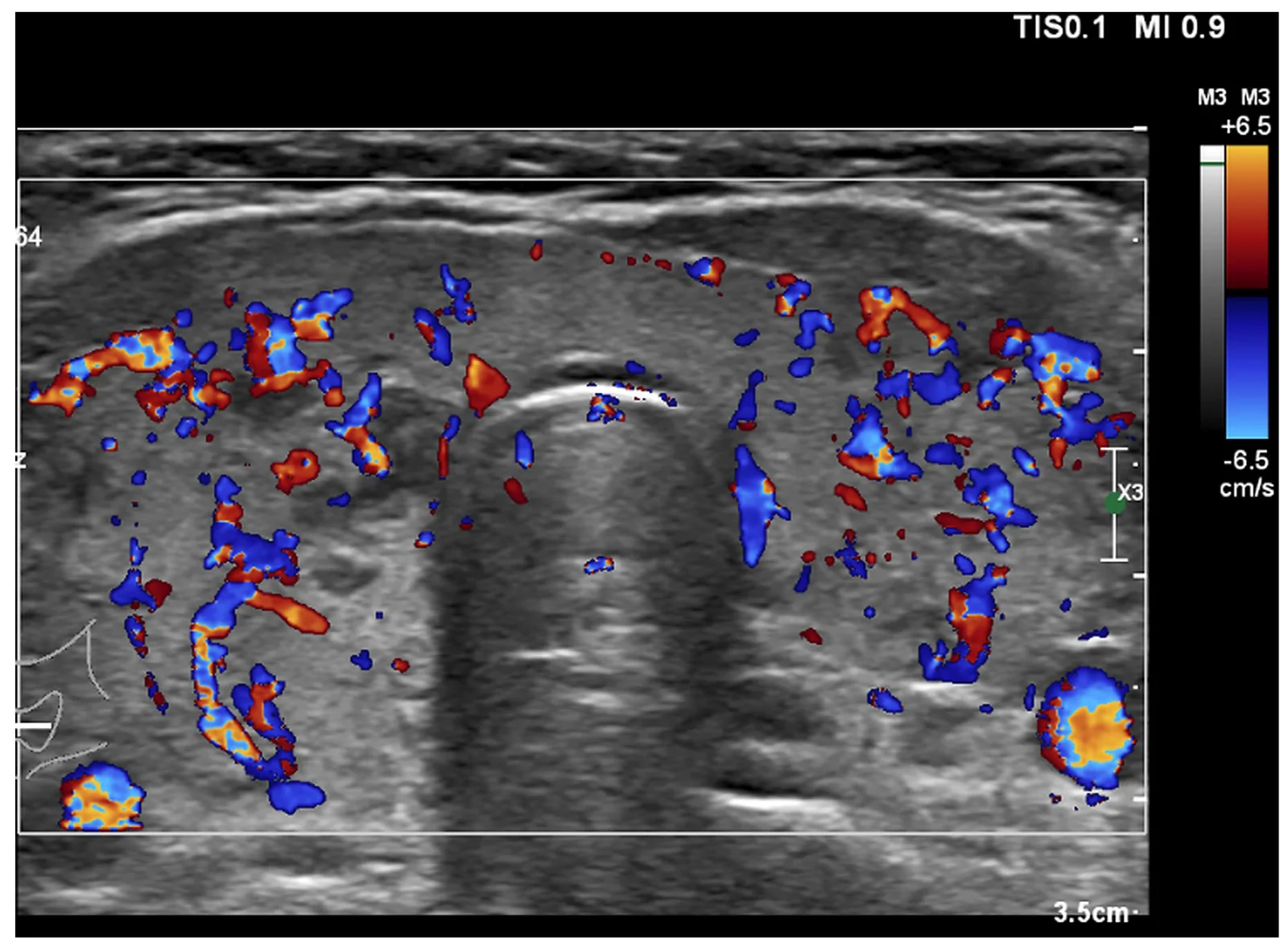
Thyroid ultrasonography shows diffuse parenchymal change with heterogeneous echotexture; no discrete nodule was identified.

With continuous targeted follow-up and nursing intervention, the patient experienced mild discomfort during the indwelling stent period with stable psychological status. She underwent a caesarean section at 37 weeks of gestation, approximately 5 months after stent placement, and delivered a pair of healthy dichorionic diamniotic twins (one boy and one girl) with birth weights of 2980 g and 2740 g. Apgar scores for the two neonates were 9 and 9 at 1 min, 10 and 10 at 5 min, and 10 and 10 at 10 min. After the puerperium period, the patient was readmitted for secondary management of residual ureteral calculus. During repeated nursing assessments, the patient reported intermittent palpitations, tachycardia, and heat intolerance after delivery. The resting heart rate was consistently above 110 beats per minute. Further laboratory testing on 7 February 2025 revealed a suppressed thyroid-stimulating hormone (TSH) level of 0.01 μIU/mL (reference range 0.55–4.78 μIU/mL), together with markedly elevated free triiodothyronine (FT3 > 20 pg/mL; reference range 2.30–4.20 pg/mL), free thyroxine (FT4 6.10 ng/dL; reference range 0.89–1.76 ng/dL), total triiodothyronine (T3 5.39 ng/mL; reference range 0.60–1.81 ng/mL) and total thyroxine (T4 17.48 μg/dL; reference range 4.50–10.90 μg/dL), consistent with thyrotoxicosis. Thyroid autoantibodies were also markedly elevated (thyroid-stimulating immunoglobulin 7.90 IU/L, ref: ≤0.55 IU/L; thyrotropin receptor antibody 11.97 IU/L, ref: ≤1.75 IU/L; thyroid peroxidase antibody 8645.3 IU/mL; ref: ≤34 IU/mL). Thyroid ultrasonography showed diffuse parenchymal change with heterogeneous echotexture, considered compatible with Hashimoto thyroiditis; no discrete nodule was identified (Figure 4). Taken together, these findings supported a diagnosis of either autoimmune thyroid disease or Graves’ disease; however, the final endocrinology record documented the diagnosis as thyrotoxicosis without further specification of the autoimmune subtype [6].

To avoid perioperative risks such as thyroid storm, the scheduled lithotripsy surgery was postponed, and the endocrinology department was consulted. Methimazole was not continued because of an adverse drug reaction; propylthiouracil (PTU) 100 mg three times daily combined with propranolol 10 mg three times daily (increased dosage to four times daily later) was therefore prescribed by the endocrinologist. Liver function was normal before PTU was started. Breastfeeding was discontinued during antithyroid treatment, and the neonates were formula-fed. During home-based antithyroid treatment, the patient developed irritability and mood fluctuations related to the inability to breastfeed and to the delayed surgery. Nurses promptly identified psychological abnormalities and provided professional psychological counseling, which improved and stabilized her emotional status.

**Figure 5:**
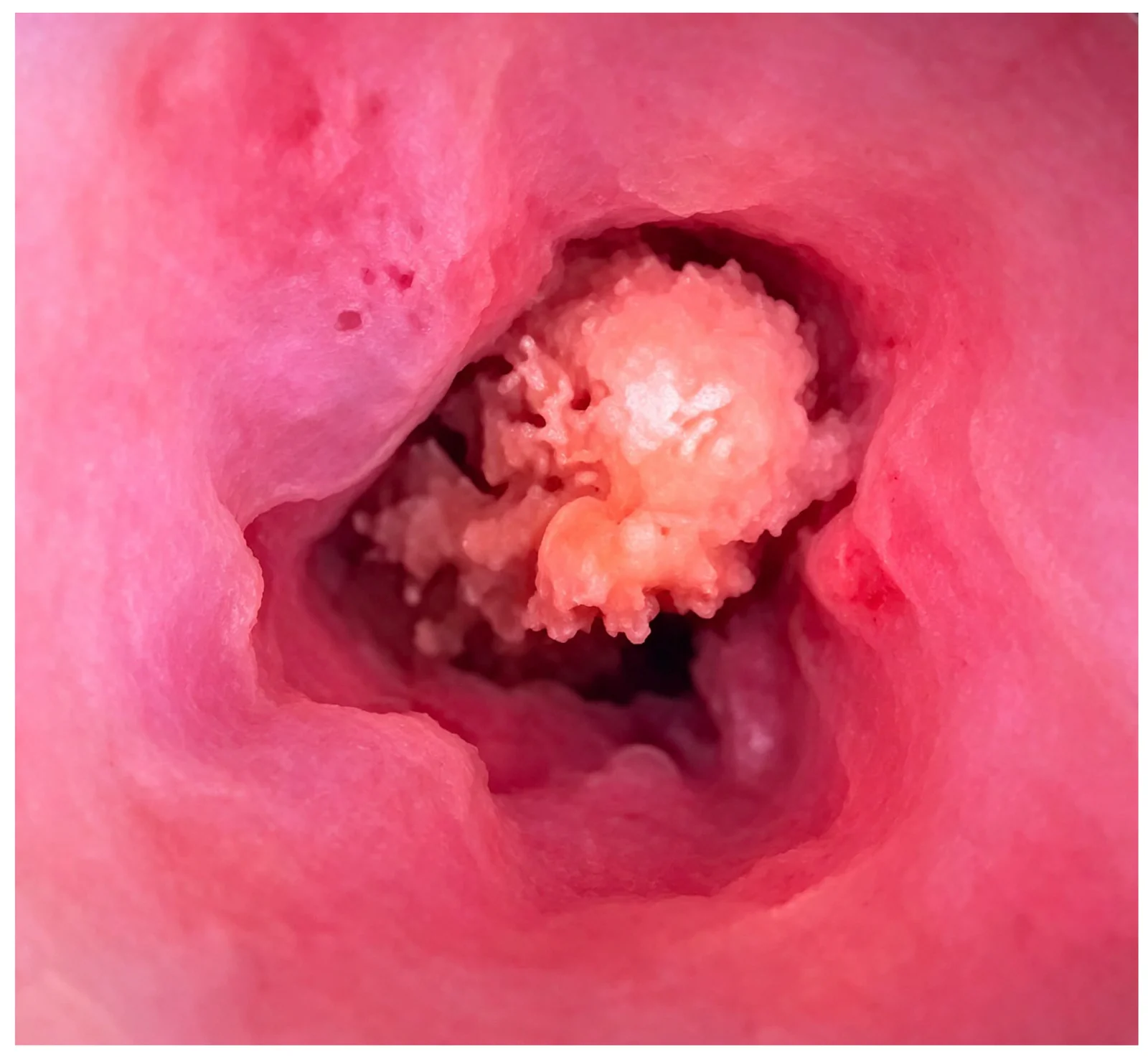
Ureteroscopic view of an ureteral calculus.

After approximately seven weeks of standardized drug therapy, follow-up on 5 March 2025 showed that TSH remained suppressed (<0.005) and FT4 remained elevated (46.73; reference range 11.92–21.62), while liver enzymes remained within the normal range and no PTU-related hepatic adverse effects were recorded. Because the patient was clinically stable, with a controlled resting heart rate and no signs of impending thyroid storm, comprehensive preoperative evaluation by the endocrinology and urology departments confirmed that she could tolerate the planned procedure, with no surgical contraindication. The patient underwent transurethral flexible ureteroscopic lithotripsy combined with ureteral stent replacement under general anesthesia (Figure 5). The operation lasted 30 min with minimal intraoperative bleeding of 1 mL. Stone component analysis confirmed a calcium oxalate monohydrate calculus. The urinary catheter and indwelling ureteral stent were removed on the third postoperative day, and the patient achieved uneventful recovery and was discharged smoothly (Table 1).

### 2.5. Patient Perspective

The patient reported that recurrent renal colic during pregnancy and concerns regarding fetal safety initially caused considerable anxiety and fear. The prolonged indwelling ureteral stent also affected her daily comfort and increased her concerns about possible complications. After thyrotoxicosis was unexpectedly identified and the planned stent removal was postponed, she experienced increased irritability, anxiety, and uncertainty regarding subsequent treatment. She considered the continuous explanations, symptom monitoring, psychological support, and online and offline follow-up provided by the nursing team helpful in improving her understanding of the disease and her confidence in treatment. Following endocrine treatment and successful stent removal, she reported substantial relief of both physical discomfort and psychological distress.

## 3. Discussion

In this case, nurse-led continuous care was implemented within a physician-directed multidisciplinary team (MDT) framework covering urology, obstetrics, endocrinology, and pharmacy. All substantive medical decisions, including the choice of conservative versus surgical management, drug selection, indications for stent placement, the antithyroid regimen, and the timing of definitive lithotripsy, were made by physicians and the MDT. Physicians from different departments took charge of specialized surgical treatment, maternal-fetal assessment, thyroid function regulation, and standardized medication management, respectively, while specially trained nurses acted as core coordinators to organize regular case discussions, adjust individualized diagnosis and nursing schemes dynamically, and conduct integrated online-offline follow-up after discharge. This collaborative mode bridges disciplinary gaps, reduces maternal-fetal perioperative risks, and realizes whole-cycle closed-loop management for complex high-risk twin pregnancy cases. In terms of disease treatment, most small-sized gestational urinary calculi can be discharged spontaneously, and conservative treatment is the preferred strategy for stable patients without severe pyogenic infection [9]. Given the patient’s 6 mm calculus, mild hydronephrosis, and absence of high fever or chills, standardized conservative treatments, including fluid infusion, early ambulation, and lateral decubitus nursing were implemented initially. A second-generation cephalosporin was administered for anti-infective treatment, while intramuscular progesterone was administered as pregnancy-supportive therapy following obstetric assessment, strictly based on gestational medication principles; NSAIDs and opioids were abandoned due to fetal adverse risks, while the application of ureteral dilating α-blockers was avoided for its unclear safety in twin gestation [10]. Once conservative treatment fails, relieving urinary tract obstruction becomes the top priority to prevent urosepsis, acute pyelonephritis, and preterm birth [11]. Compared with percutaneous nephrostomy limited by gestational body position and laser lithotripsy requiring general anesthesia, indwelling ureteral stent placement under local anesthesia was determined to be the optimal option for this patient, as this minimally invasive method avoids general anesthesia-related fetal hazards and rapidly alleviates recurrent renal colic, matching the clinical features of high-risk twin pregnancy complicated with thyrotoxicosis.

**Figure 6:**
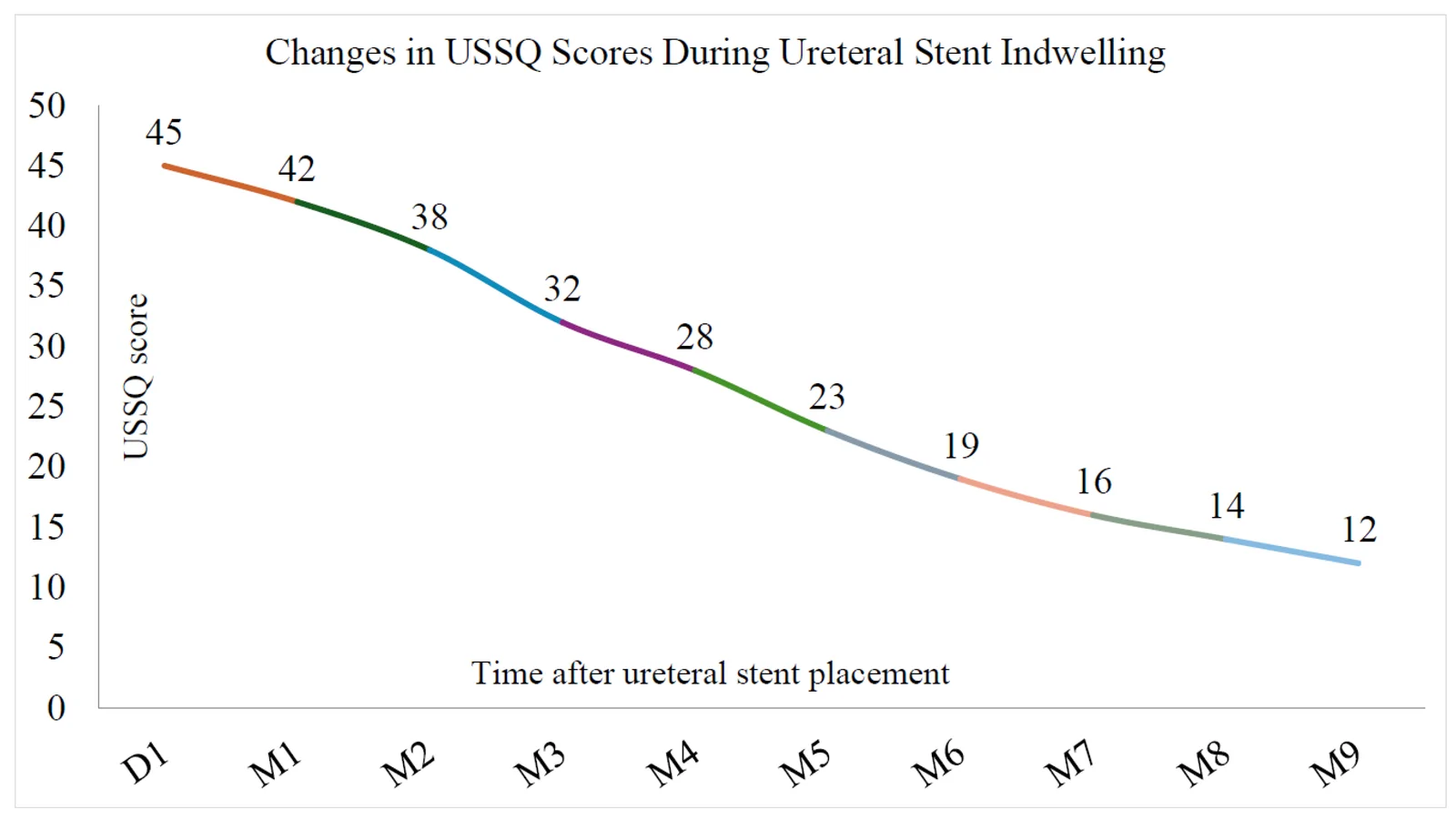
Changes in Ureteral Stent Symptom Questionnaire (USSQ) scores from ureteral stent placement to removal. X axis: D1, day 1; M1–M9, months 1–9 after ureteral stent placement. Y axis: total USSQ score. Lower scores indicate fewer stent-related symptoms and greater comfort. Data are serial, self-reported measurements obtained from a single patient.

As common implanted devices in urology, ureteral stents can relieve urinary tract obstruction and prevent ureteral stricture, yet the indwelling foreign body is prone to cause urinary infection, lumbago, and crystal deposition during long-term retention [12,13]. Such persistent stent-related symptoms further trigger negative emotions and reduce quality of life among pregnant women. In this case, the Ureteral Stent Symptom Questionnaire was adopted to quantitatively evaluate stent-related complications so as to formulate targeted home nursing guidance. Regular monitoring of urine characteristics, standardized water intake guidance, and graded activity intervention lowered the incidence of stent-related adverse events during the nine-month indwelling period (Figure 6). Notably, conventional internet-based nursing merely provides one-way health education and simple remote consultation without a multidisciplinary risk early warning mechanism, resulting in poor patient self-management compliance. Therefore, this study optimized the online nursing model by integrating remote maternal-fetal monitoring, dynamic symptom assessment, and MDT joint intervention; unlike single-mode online follow-up, this integrated model achieves risk identification and individualized intervention and further improves home nursing and treatment satisfaction for discharged pregnant patients with indwelling stents. The stent used in this case was a 4.8 French gauge (Fr) ureteral stent (diameter 1.6 mm, length 26 cm, Percuflex™ Plus Ureteral Stent with HydroPlus™ Coating, M0061752530. Marlborough, MA, USA) designed for long-term indwelling, with a maximum recommended indwelling time of up to 365 days (as stated in the instructions) [14]. The risks of prolonged stent indwelling, including encrustation, urinary tract infection, obstruction, and the possible need for further intervention, were fully explained to the patient. Because she was reluctant to undergo repeated invasive procedures during the twin pregnancy, she declined stent exchange before delivery. After multidisciplinary assessment and shared decision-making, her informed choice was respected, and no clinical indication for urgent stent exchange was identified before delivery. Nurse-led e-health interventions have been reported to support self-management and health-related outcomes in pregnant women [15].

Pregnant patients with urinary calculi have a high incidence of anxiety, especially young women with twin gestation [16,17]. In this case, the patient’s negative emotions mainly derived from fetal safety concerns, stent-induced physical discomfort, and thyroid-related mood fluctuation, as well as daily life and work pressure (Figure 3).

Accordingly, a combined intervention including online psychological counselling, music therapy, and hierarchical health education was delivered throughout hospitalization and follow-up, consistent with evidence that internet-delivered and psychoeducational interventions can reduce perinatal anxiety [18,19,20], and emotional intervention stabilized her mood after delayed lithotripsy surgery and boosted long-term treatment compliance. Clinically, twin pregnancy combined with thyrotoxicosis is classified as a high-risk gestational complication, which may induce thyroid storm, gestational hypertension, and adverse pregnancy outcomes without standardized intervention. For this patient, propylthiouracil was applied to stabilize thyroid function with negligible fetal adverse effects. Combined with perioperative vital sign monitoring, heart rate management, and emotional nursing, the patient maintained stable thyroid metabolism and physical condition during secondary lithotripsy, without thyroid crisis, preterm labor, or other critical complications. Stone component analysis in this case identified calcium oxalate monohydrate. Pregnancy-related anatomical and urinary changes may have contributed to stone formation [5], while hypercalciuria is an established metabolic risk factor for calcium-containing calculi. Excess thyroid hormone may theoretically accelerate bone turnover and calcium mobilization [21]; however, because thyroid dysfunction was detected several months after the initial stone event, the present case does not establish a causal association between the two conditions. The favourable outcome in this case should not be attributed to nursing intervention alone. Rather, it resulted from the combined contributions of urologists, obstetricians, endocrinologists, pharmacists, and nurses, with nurses primarily providing continuous assessment, care coordination, patient education, and follow-up. These core elements, including nurse-led assessment, predefined escalation criteria, and multidisciplinary communication, may provide a practical basis for the future development of a standardized nursing care pathway for similar patients [22]. Admittedly, this is a single-case retrospective report without a comparator, and larger prospective studies are required to verify the applicability of this care model.

## 4. Conclusions and Prospect

Standardized, high-quality nursing management is crucial for pregnant women diagnosed with symptomatic urinary calculi. Standardized holistic nursing can facilitate maternal physical rehabilitation, relieve peripartum negative emotions, optimize medical experience, and improve long-term quality of life. For this complex high-risk gestational case featuring acute renal colic, dichorionic diamniotic twin pregnancy, secondary urinary tract infection, and concurrent thyrotoxicosis, nurse-led continuous care implemented within a physician-directed multidisciplinary framework enabled precise condition assessment, early maternal-fetal risk identification, and uninterrupted whole-cycle nursing intervention. Integrated interventions including standardized gestational analgesia, evidence-based anti-infective treatment, standardized perioperative monitoring, internet-based home continuous care, and targeted psychological support contributed to favorable maternal-fetal outcomes in this case. This case illustrates a feasible approach to nurse-led continuous care within a physician-directed multidisciplinary framework for a patient with complex pregnancy-related and subsequent endocrine conditions. However, as this is a single case, the findings should be interpreted cautiously and require further validation in larger prospective studies.

## Figures and Tables

**Table 1:** Timeline of clinical events, management, and outcomes.

Date	Clinical Event	Key Management/Outcome
27 June 2024	Emergency admission at 14 + 3 weeks of twin gestation with right renal colic; 6 × 6 mm right ureteral calculus with mild hydronephrosis	Ureteral stent placement under local anaesthesia; discharged on postoperative day 2
July–September 2024	Internet-based continuing care during stent indwelling	Monthly USSQ and HADS-A assessment; health education and follow-up
30 September 2024	Urology follow-up	Continued surveillance during stent indwelling
November 2024	Caesarean section at 37 weeks of gestation	Healthy boy-girl twins delivered (2980 g and 2740 g; Apgar 9/9, 10/10, 10/10)
20 January 2025	Urology follow-up	Residual stone and stent status reassessed
27 January 2025	Urology follow-up	Flexible ureteroscopic lithotripsy scheduled
6 February 2025	Admission for planned surgery; persistent tachycardia detected preoperatively	Surgery postponed; endocrine assessment initiated
7–8 February 2025	Thyroid function tests, thyroid autoantibodies, and thyroid ultrasonography	Thyrotoxicosis diagnosed; propylthiouracil and propranolol started; breastfeeding discontinued
February–March 2025	Continued endocrine treatment at an outside hospital	Serial thyroid function monitoring and treatment optimization
5 March 2025	Endocrinology follow-up	Perioperative thyroid status reassessed; no contraindication to surgery
28 March 2025	Flexible ureteroscopic lithotripsy	Stone cleared (calcium oxalate monohydrate); ureteral stent removed after 9 months of indwelling

## Data Availability

All relevant data are included within the article.
